# Physicochemical Characterization and In Vitro Biocompatibility of Epoxy- and Silicone-Based Endodontic Materials

**DOI:** 10.3390/ma19071388

**Published:** 2026-03-31

**Authors:** Alexandra Popa, Marina Imre, Silviu Mirel Pițuru, Bianca Voicu-Bălașea, Ana Cernega, Radu-Vasile Rădulescu, Florentina Rus, Roxana Trușcă, Monica Musteanu, Ecaterina Andronescu, Alexandra Ripszky

**Affiliations:** 1 The Interdisciplinary Center for Dental Research and Development, Faculty of Dental Medicine, “Carol Davila” University of Medicine and Pharmacy, 19-21 Jean Louis Calderon Street, 020021 Bucharest, Romaniamarina.imre@umfcd.ro (M.I.); ana.cernega@umfcd.ro (A.C.); florentina.rus-hrincu@umfcd.ro (F.R.); alexandra.ripszky@umfcd.ro (A.R.); 2 Department of Biochemistry, Faculty of Dental Medicine, University of Medicine and Pharmacy Carol Davila, 37 Dionisie Lupu Street, District 2, 020021 Bucharest, Romania; radu.radulescu@umfcd.ro; 3 Department of Complete Denture, Faculty of Dental Medicine, University of Medicine and Pharmacy Carol Davila, 17-23 Calea Plevnei, 010221 Bucharest, Romania; 4 Department of Professional Organization and Medical Legislation-Malpractice, “Carol Davila” University of Medicine and Pharmacy, 37 Dionisie Lupu Street, District 2, 020021 Bucharest, Romania; 5 National Research Center for Micro and Nanomaterials, National Polytechnic University of Science and Technology of Bucharest, 060042 Bucharest, Romania; roxana_doina.trusca@upb.ro (R.T.); ecaterina.andronescu@upb.ro (E.A.); 6 Department of Biochemistry and Molecular Biology, Faculty of Pharmacy, University Complutense of Madrid, 28040 Madrid, Spain; 7 Department of Science and Engineering of Oxide Materials and Nanomaterials, Faculty of Chemical Engineering and Biotechnologies, National Polytechnic University of Science and Technology of Bucharest, 011061 Bucharest, Romania; 8 Romanian Academy of Scientists, 050045 Bucharest, Romania

**Keywords:** endodontic sealers, biocompatibility, X-Ray diffraction, FT-IR, particle size

## Abstract

Root canal sealers play a crucial role in the success of endodontic treatment, facilitating healing and regeneration of the periapical region. This study aimed to evaluate the biological, physicochemical and structural properties of two sealers, AH Plus and ROEKO GuttaFlow 2. Scanning electron microscopy (SEM) analysis reveals polyhedral particles uniformly distributed within the porous organic matrix of AH Plus, whereas ROEKO GuttaFlow 2 exhibits a heterogeneous structure, with particles distributed evenly. Fourier-Transform Infrared Spectroscopy (FT-IR) analysis confirmed the characteristic chemical bonds associated with both the organic and inorganic phases of each material, while X-Ray diffraction analysis identified the main crystalline phases (CaWO_4_ and ZrO_2_ for AH Plus and ZrSiO_4_ and ZrO_2_ for ROEKO GuttaFlow 2). The biocompatibility tests were performed on human osteosarcoma cells (ATCC—G 292 CRL-1423). An in vitro metabolic activity and viability test (MTT) showed a significant decrease by ~92% (* *p* < 0.05) and ~87% after 24 and 48 h for samples incubated with AH Plus versus the control. Regarding ROEKO GuttaFlow 2, MTT levels increased by ~8% in the first 24 h, while after 48 h they decreased by ~11% versus control. Lactate dehydrogenase levels significantly increased at 24 and 48 h for cells incubated with AH Plus (*** *p* < 0.001, ** *p* < 0.01). ROEKO GuttaFlow 2 significantly decreased the LDH level at 24 h (** *p* < 0.01), while at 48 h a rise was observed. The significantly increased levels of nitric oxide observed in cells incubated with the materials at 24 and 48 h (** *p* < 0.01, *** *p* < 0.001) suggest a cellular adaptation to our experimental environment. Overall, ROEKO GuttaFlow 2 exhibited a more favorable profile under our testing conditions.

## 1. Introduction

Endodontics focuses on preserving teeth with pulp or periapical damage on the dental arch through treatments aimed at eliminating infection and ensuring a tight three-dimensional seal of the endodontic system [1,2]. Besides the operative technique, the long-term success of root canal treatments and apical surgeries is also influenced by the quality of the materials used [3,4].

The emergence of new types of endodontic sealants is driven by the need for materials that accelerate healing and tissue regeneration [5,6,7]. Although these materials offer superior physicochemical properties and biocompatibility, certain limitations may remain. Issues related to dimensional stability may persist, compromising tightness as microgaps appear [8]. When extruded into the periapical region, the sealant may cause local inflammatory responses. In this context, the material can interact with fibroblasts, cementoblasts, osteoblasts, stem cells, periodontal ligament cells, endothelial cells, and immune cells, such as B and T lymphocytes and macrophages [9,10,11,12]. Activation of these immune cells results in the release of proinflammatory cytokines (TNF-α, IL-1β, IL-6, IL-18, MIP-1, MCP-1) and receptor activator of nuclear factor-κB ligand (RANKL), thereby promoting osteoclastogenesis and subsequent bone resorption [13,14,15]. Thus, the regeneration of bone tissue after endodontic treatment, which relies on osteoblast activity, indicates the success of the procedure [16,17,18]. Moreover, nitric oxide (NO), produced by cells in response to stimuli, is a key mediator of inflammation and plays a crucial role in activating cellular pathways that contribute to periapical tissue inflammation, post-operative symptoms, and delayed healing [19,20].

A significant part of the research in the field of endodontics focuses on developing materials that not only seal the canal and promote bone regeneration but also exhibit high biocompatibility and optimal physicochemical properties. Thus, according to the definition accepted and used since 1987, “Biocompatibility refers to the ability of a material to perform with an appropriate host response in a specific situation”. To fulfil this desire, materials have been developed based on a list of negative requirements: non-toxic, non-irritant, non-carcinogenic, non-hemogenic, non-teratogenic and so on [21,22].

The most used materials, based on their main components, include calcium hydroxide, epoxy and methacrylic resins, zinc oxide eugenol, calcium silicate, silicones, and glass ionomer cements, each with its own properties, advantages, and disadvantages [23,24,25]. Even if some modern endodontic materials are designed to support tissue regeneration and encourage the healing of periapical tissues, no product possesses all ideal characteristics, particularly in terms of biocompatibility, which is important given the prolonged contact they have with the periodontium [26,27,28].

Over time, AH has undergone several compositional changes to develop a more tissue-friendly formulation. Although mechanically efficient, AH 26, one of the first epoxy resin sealants introduced in the 1970s, released formaldehyde immediately after curing [29,30,31,32]. This issue led to a redesign of the material’s chemical composition. Currently, although AH Plus no longer releases formaldehyde and features improved physical properties such as low solubility and minimal dimensional changes compared to other sealers, clinically relevant properties as they contribute to long-term sealing capacity and stability of the root canal obturation, better flow and shorter setting time, its cytotoxicity remains, most likely due to the amines acting as accelerators of the setting reaction [33,34,35].

ROEKO GuttaFlow 2 is an innovative cold obturation material with a fluid consistency. The product combines gutta-percha powder with particles smaller than 30 μm, polydimethylsiloxane, and zirconium dioxide in a single system. The main advantage of this material is given by its fluidity, low solubility and expansion capacity during the setting time, thus ensuring optimal sealing of the root canal [35,36,37].

AH Plus and ROEKO GuttaFlow 2 are widely used endodontic sealers with distinct chemical compositions, which may influence their physicochemical properties and behavior. Therefore, a comparative evaluation of these materials is necessary for a better understanding of their performance and biocompatibility.

Currently, the literature provides limited data on the biocompatibility of endodontic sealing materials on human osteoblasts and osteosarcoma cells. The assessment of cell cytotoxicity is important, as these materials can come into contact with periapical bone, and osteoblasts are the key cells responsible for the tissue response in this area. Analysis of the effects of sealants on osteoblast viability, proliferation, differentiation, and mineralization provides mechanistic information about the possible impact on periapical bone healing and regeneration. Moreover, toxic effects on osteoblasts may alter cytokine signaling and the RANKL/OPG balance, promoting osteoclastogenesis and bone loss [38,39].

Therefore, in this research, we aimed to evaluate the biocompatibility of certain endodontic materials with human osteosarcoma cells (G292 cells), along with their physicochemical properties. The study involved two types of endodontic materials from different classes: AH Plus, an epoxy resin sealer, and ROEKO GuttaFlow 2, a silicone-based material.

## 2. Materials and Methods

Two types of endodontic materials from two different classes have been selected for this study. The materials examined in this study were AH Plus (Dentsply Sirona, DeTrey GmbHb De-Trey-Str.1 78467 Konstanz, Germany), an epoxy resin sealer, and ROEKO GuttaFlow 2 (Coltene/Whaledent AG, Altstätten, Switzerland), a silicone-based sealer (Table 1).

### 2.1. Sample Preparation

From each material were processed 39 samples (discs with 7 mm diameter and 2 mm thickness) using polyvinyl chloride molds and allowed to set at 37 °C with 5% CO_2_ and 95% humidity for 24 h (AH Plus) and 30 min (ROEKO GuttaFlow 2), in accordance with the manufacturer’s instructions [40,41]. AH Plus was mixed with a sterile spatula on a sterile glass plate and then transferred into the matrix (mixing ratio paste A: paste B—1:1), while ROEKO GuttaFlow 2 was directly injected into the molds using mixing tips from the manufacturer (mixing ratio 4:1). Subsequently, the samples were exposed to UV light for 10 min on each side to minimize the possibility of microbial contamination of cells. After this time interval, the samples were immediately subjected to testing, as follows: 30 discs were used for MTT, LDH and NO assays, 5 discs for EDX and 1 disc each was allocated for SEM, FT-IR and XRD analyses. The additional disc was allocated for SEM analysis at 14 days post-setting, during which it was stored in an incubator at 37 °C with 5% CO_2_ and 95% humidity until examination.

### 2.2. Morphological and Structural Characterization

#### 2.2.1. Scanning Electron Microscopy (SEM) and Energy Dispersive X-Ray Spectrum (EDX)

SEM analysis was performed with a QUANTA INSPECT F50 microscope from Thermo Fisher, Eindhoven, the Netherlands. The microscope features an energy dispersive X-Ray spectrometer (EDX), which allows elemental analysis with a resolution of 133 eV at the Mn Kα line. The microscope also integrates a field-emission electron gun (FEG), capable of reaching a resolution of 1.2 nm, facilitating detailed nanoscale images. To ensure good electrical conductivity, SEM samples were sputtered with gold for 60 sec using the Q150R Plus Sputter Coater/Evaporator system—Quorum Technologies, East Sussex, UK. After coating, the samples were inserted into the microscope’s analysis chamber, and images were obtained by collecting secondary electrons emitted from the materials’ surfaces, revealing detailed information about particle morphology and size. The EDX samples were examined separately and were not coated with gold to avoid interference with elemental quantification and mapping.

#### 2.2.2. Fourier-Transform Infrared Spectroscopy (FT–IR)

The analysis of the functional groups of the selected endodontic materials was carried out using a Thermo Nicolet 6700 FT-IR spectrometer (Thermo Fisher Scientific, Waltham, MA, USA), equipped with a ZnSe crystal. The measurements were carried out at room temperature, using 32 scans per sample to ensure reproducibility and accuracy of the results. Spectral data were recorded with a resolution of 4 cm^−1^, over the range 4000–400 cm^−1^. The instrument was operated using Omnic32 software (Thermo Nicolet, v.8.2-Thermo Fisher Scientific, Waltham, MA, USA), connected to a processing system and a database, enabling the acquisition and precise interpretation of IR spectra.

#### 2.2.3. X-Ray Diffraction (XRD)

The crystalline phases of the selected materials were identified by XRD analysis, using a PANalytical Empyrean diffractometer (Malvern PANalytical, Bruno, the Netherlands), equipped with a hybrid monochromator (2xGe 220) on the incident beam, which ensured precise control of the X-radiation. On the diffracted side, detection was performed using a parallel plate collimator installed on a three-dimensional (3D) PIXcel detector, optimizing the accuracy of the measurements. Experiments were performed at room temperature, with an incidence angle of 0.5°, over a Bragg 2θ angle range from 10° to 80°. Collection times were set to 255 s per acquisition, with a step size of 0.01414°, to provide accurate information. The radiation source used was Cu Kα (λ = 1.5406 Å), operated at 40 mA and 45 kV.

### 2.3. Metabolic Activity, Viability and Cytotoxicity

#### 2.3.1. Cell Culture

Human osteosarcoma cells (G 292 CRL-1423—ATCC, 10801 University Boulevard Manassas, VA 20110-2209 USA) [42] were cultured in 75 cm^2^ culture flasks using Dulbecco’s modified Eagle medium (DMEM)/F12 (Sigma-Aldrich^®^ Solutions, Merck KGaA, Darmstadt, Germany) supplemented with 10% fetal bovine serum and 1% antibiotic and antifungal. The medium was changed every 3 days, and the flasks were stored in a humidified incubator with 5% CO_2_ at 37 °C.

For the experiments, the cells (G292) were seeded at a density of 10^4^ cells per well in a 24-well plate with 500 μL DMEM/F12 medium and allowed to adhere overnight. Subsequently, the material samples were added over the monolayer cells and incubated for 24 and 48 h with 5% CO_2_ at 37 °C. After the incubation period, the materials were removed, and the appropriate tests were subsequently performed. The control consisted of cells grown in DMEM/F12 medium without any dental material. The cells were placed in direct contact with the material because, clinically, when the material is extruded, it comes into direct contact with periradicular tissue cells (periodontal ligament cells, osteoblasts, etc.) [10].

#### 2.3.2. Metabolic Activity and Viability Assay

To perform the metabolic activity and viability assay, the MTT solution was used. A 3-(4,5-dimethylthiazol-2-yl)-2,5-diphenyltetrazolium bromide assay (MTT; Sigma-Aldrich, Darmstadt, Germany) was performed at a concentration of 1 mg/mL. The underlying principle is that only metabolically active cells can convert MTT into formazan, which appears violet [43] and is measured spectrophotometrically at a wavelength of 595 nm using a FLUOstar^®^ Omega multi-mode microplate reader (BMG LABTECH, Ortenberg, Germany). The test was performed in triplicate (*n* = 3), according to the manufacturer’s instructions.

#### 2.3.3. Level of Lactate Dehydrogenase

The reaction principle involves measuring the level of the enzyme lactate dehydrogenase (LDH) released into the culture medium after the cell membrane has lost its integrity. With NAD+ as a cofactor, LDH catalyzes the conversion of L-lactate into pyruvate. Thus, NAD+ is reduced to NADH during this process [44].

The LDH levels were determined using the LDH Cytotoxicity Assay (LDH, Thermo Fisher Scientific, Eugene, OR 97402 USA) REF: C20300, following the manufacturer’s protocol, in six replicates (*n* = 6). Supernatants were transferred to another plate for reaction with the LDH mix, and absorbance was measured after 30 min at 540 nm using the FLUOstar^®^ Omega multi-mode microplate reader (BMG LABTECH, Ortenberg, Germany).

#### 2.3.4. Level of Nitric Oxide (NO)

To assess nitric oxide levels, the Nitric Oxide Assay Kit (Abnova, Taipei City, Taiwan), REF: KA1641, was employed, which detects nitrates and nitrites in the culture medium. Nitrates are reduced to nitrites by NADPH in the presence of the enzyme nitrate reductase. The resulting nitrites then react with sulfanilamide and N-(1-naphthyl)-ethylenediamine dihydrochloride, forming a red–violet compound. As a result, the nitrite concentration indicates the nitric oxide level [45]. Supernatants were transferred to another plate for reaction with the Griess reagents. Quantification of the results was performed spectrophotometrically after 10 min, at 60°, at 540 nm using the FLUOstar^®^ Omega multi-mode microplate reader (BMG LABTECH, Ortenberg, Germany). The test was repeated six times (*n* = 6).

### 2.4. Statistics

The results were reported compared to the control wells (100%) and displayed visually. The graphs were created using the arithmetic means of results for each test type, and variability was expressed as mean ± standard deviation (SD), performed with Microsoft Office Excel (Microsoft Corporation One Microsoft Way, Redmond, WA 98052-6399, USA). Statistical analysis was performed using IBM Statistical Package for the Social Sciences (SPSS), version 26.0 (IBM—New York, NY, USA). The distribution of quantitative variables was assessed by the Shapiro–Wilk test, and the homogeneity of variances by the Levene test. Analyses were performed separately for each time point (24 and 48 h), comparing the two groups (AH Plus and ROEKO GuttaFlow 2) with the control.

For data with a normal distribution, differences between groups were analyzed using one-way analysis of variance (one-way ANOVA) followed by the Games–Howell post hoc test. In case of violation of homogeneity of variances, Welch ANOVA was applied, followed by the Games–Howell post hoc test. For data that did not respect the assumptions of normality, the Kruskal–Wallis H test was used, and post hoc comparisons were performed by the Mann–Whitney U test, followed by the application of the Bonferroni correction for multiple comparisons (adjusted α = 0.05/3). The statistical significance threshold was set at α = 0.05 or adjusted α when the Bonferroni correction was applied.

## 3. Results

### 3.1. AH Plus

#### 3.1.1. Surface Characterization and Chemical Composition

**Figure 1:**
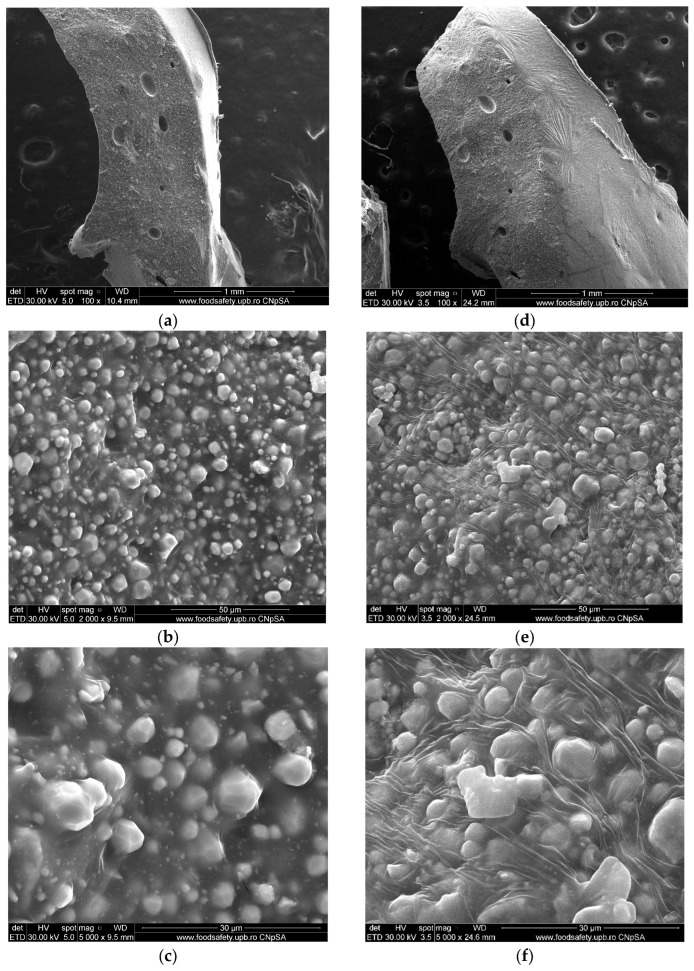
SEM micrography recorded for AH Plus. At 24 h after setting, polyhedral particles well integrated in the organic matrix and pores are visible at magnifications of (**a**) 100×, (**b**) 2000× and (**c**) 5000×. The outer surface (**a**) exhibits microtextural features. After 14 days, the outer, microtextured, surface of the sample presents fine lines arranged radially, resembling a star shape at magnification of (**d**) 100×. Inside, the resin forms a wavy system that covers the polyhedral granules, as seen at 2000× (**e**) and 5000× (**f**).

Twenty-four hours after setting, SEM images taken at different magnifications (100×, 2000×, 5000×) (Figure 1a–c) reveal polyhedral particles, most likely inorganic compounds, uniformly distributed within the organic matrix represented by epoxy resin. The section surface appears relatively compact, with the inorganic phase well embedded in the organic matrix. Noticeable porosities are visible inside (Figure 1b,c) and on the outer, microtextured, surface (Figure 1a) of the sample in the organic matrix, most likely caused by inhomogeneities during the material’s preparation.

After 14 days, fine lines, arranged in a star shape, can be observed on the outer, unevenly structured surface of the sample (Figure 1d). These may occur due to late polymerization shrinkage of the material. Inside, the resin sets in a wavy system, covering the ceramic granules and “wetting” their surface (Figure 1e,f). This aspect highlights the good compatibility of the organic resin with the inorganic granules. However, the resin shows different behavior between the large granules, on which it adheres, and the small granules, which it does not stick to, but rather encapsulates.

**Figure 2:**
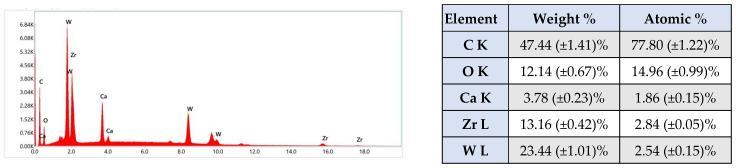
Qualitative and quantitative energy dispersive X-Ray spectrum associated with AH Plus. The results are illustrated as mean ± SD (*n* = 5).

Qualitative and quantitative analysis using EDX spectrum confirms the presence of inorganic chemical compounds, Zr, Ca and W (Figure 2). In the EDX spectrum, the symbols Kα and Lα represent the type of electronic transition detected for each chemical element. Light elements (O, C, Ca) show Kα lines, while for heavy elements (Zr, W) Lα lines are mainly detected, due to the high energy of the Kα lines [46].

**Figure 3:**
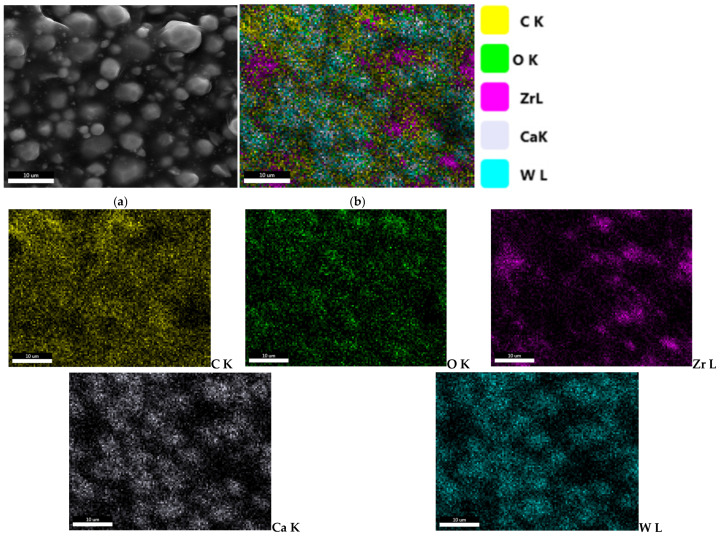
Elemental mapping recorded for AH Plus. Backscattered electron images (**a**) and surface distribution images (**b**) of the relative intensity of X-Ray radiation specific to the significant elements detected, C Kα, O Kα, Zr Lα, Ca Kα, W Lα, on the microarea of the AH Plus mixture sample. The images highlight the variable distribution of the chemical elements, from uniform, homogeneous regions to concentrated areas.

EDX mapping of the AH Plus material highlights the presence of the elements C, O, Zr, Ca and W with variable distributions, from uniform, homogeneous regions to concentrated areas (Figure 3). This suggests the existence of crystalline phases, CaWO_4_ and ZrO_2_, which were subsequently identified by diffraction.

#### 3.1.2. FT-IR AH Plus

**Figure 4:**
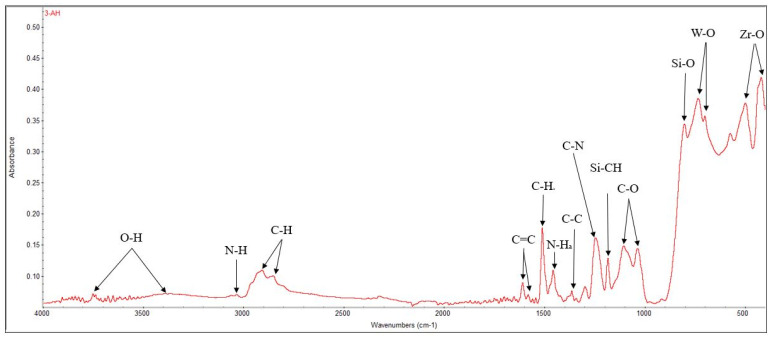
FT-IR spectra recorded for AH Plus.

FT-IR spectroscopy was recorded in the range of 4000–400 cm^−1^, with emphasis on the final region, the fingerprint, specific to the characteristic vibrations of metal–oxygen bonds and complex inorganic groups. Thus, at wavelengths of 496 and 440 cm^−1^, Zr-O bonds are highlighted. At 731 and 698 cm^−1^, W-O bonds can be identified, attributed to the tungstate ion and explained by the presence of calcium tungstate in the composition of the material. Also, bonds with silicon can be identified, respectively, Si-O at 800 cm^−1^ and Si-CH at 1181 cm^−1^. The presence of C-N bonds at 1243 cm^−1^ and N-H bonds at wavelengths of 1454 cm^−1^ and 3030 cm^−1^ is indicative of nitrogen compounds, specifically amines, which serve as strengthening agents (Figure 4) [47,48].

#### 3.1.3. XRD AH Plus

**Figure 5:**
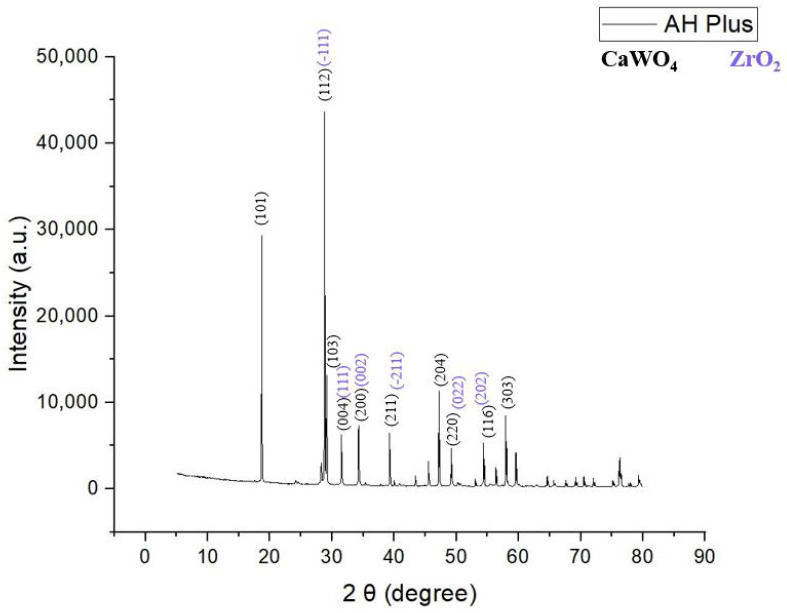
XRD analysis recorded for AH Plus.

Following XRD analysis of the AH Plus material, two crystalline phases were identified: tetragonal calcium tungstate/scheelite (CaWO_4_—ASTM 01-083-5705) [49] and monoclinic zirconium oxide (ZrO_2_—ASTM 01-075-6808) [50]. The diffraction intensity maxima recorded at 43,623 and 2θ = 28.803° and positioned at the Miller indices (112) for CaWO_4_ and at 22,743 and 2θ = 28.777°, positioned at (−111) for ZrO_2_, indicate a high degree of crystallinity of the material, according to ASTM data sheets (Figure 5) [49,50]. Table 2 and Table 3 summarize the correlations between the ASTM reference (hkl, d, 2θ, I%) and the experimentally measured diffraction angles (2θ) and d-spacing.

### 3.2. ROEKO GuttaFlow 2

#### 3.2.1. Surface Characterization and Chemical Composition

**Figure 6:**
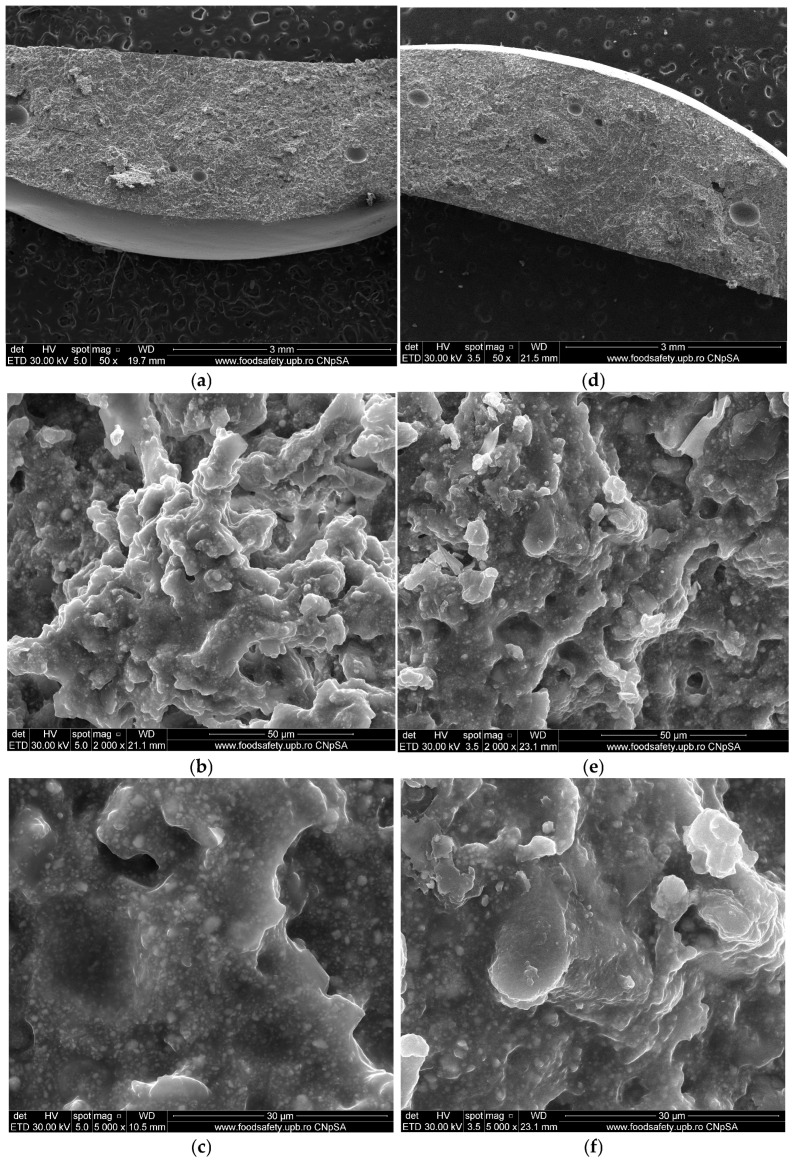
SEM micrography recorded for ROEKO GuttaFlow 2. Thirty minutes after setting, the material reveals a smooth, non-porous outer surface at (**a**) 50×. At higher magnifications of (**b**) 2000× and (**c**) 5000×, the material section exhibits visible pores and granules, with small particles uniformly distributed within the matrix. After 14 days, no significant morphostructural changes were evident compared to samples analyzed 30 min after setting, at magnifications of (**d**) 50×, (**e**) 2000× and (**f**) 5000×.

At 30 min post-setting, at 50× magnification (Figure 6a), the outer surface of the material appears smooth and free of porosity. Increasing the magnification to 2000× and 5000× (Figure 6b,c) reveals that the section of the material is relatively heterogeneous, with identifiable pores and granules on the surface and small particles uniformly embedded within the structure. Thread-like structural elements are visible inside the material, which may be attributed to the fact that the images were taken on the section, and mechanical artefacts may have appeared during fracturing. The heterogeneity is further supported by images at higher magnifications, where pores and cavities can be identified inside the silicone matrix. The inhomogeneities seem to be generated by the organic phase, which is also responsible for the formation of cavity-type porosities, while the inorganic, crystalline phase is evenly distributed. No morphological or structural changes were observed 14 days after setting, as assessed by SEM analysis (Figure 6d–f). The microstructure remained consistent with that observed immediately after setting, indicating that the material maintained its integrity over the studied period and did not undergo significant microstructural modifications.

**Figure 7:**
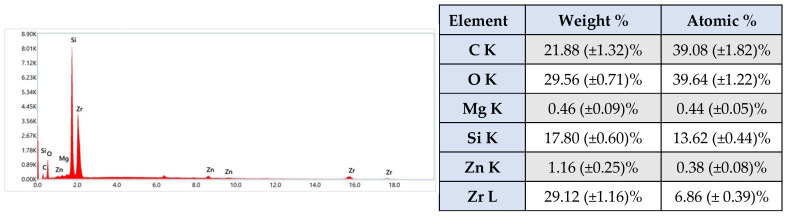
Qualitative and quantitative energy dispersive X-Ray spectrum associated with ROEKO GuttaFlow 2. The results are illustrated as mean ± SD (*n* = 5).

EDX analysis (Figure 7) confirms the presence of the inorganic elements found in the substances that compose the material, through the presence of the chemical elements Si and Zr. Also, secondary elements, Zn and Mg, were identified following the analysis of the material. In this case, the presence of zinc can be justified by its antibacterial and antifungal properties [51].

**Figure 8:**
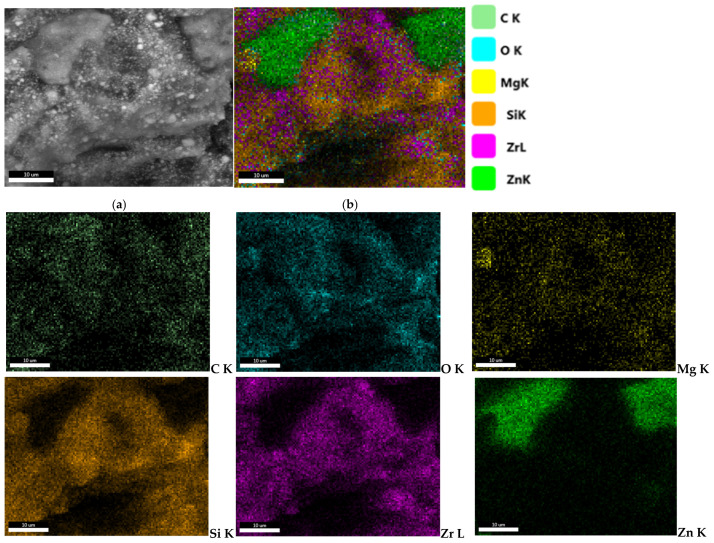
Elemental mapping recorded for ROEKO GuttaFlow 2. Backscattered electron images (**a**) and surface distribution images (**b**) of the relative intensity of X-Ray radiation specific to the significant elements detected, C Kα, O Kα, Mg Kα, Si Kα, Zr Lα, Zn Kα on the microarea of the ROEKO GuttaFlow 2 mixture sample.

Elemental mapping reveals the precise distribution of elements within the sample. Therefore, inside the ROEKO GuttaFlow 2 sample, Zr, Si, Mg, C and O can be observed with a relatively uniform distribution in well-defined areas, while Zn crystals seem to be concentrated between the spaces left free by the other elements that form ZrO_2_ and ZrSiO_4_ (Figure 8), subsequently identified by XRD analysis.

#### 3.2.2. FT-IR

**Figure 9:**
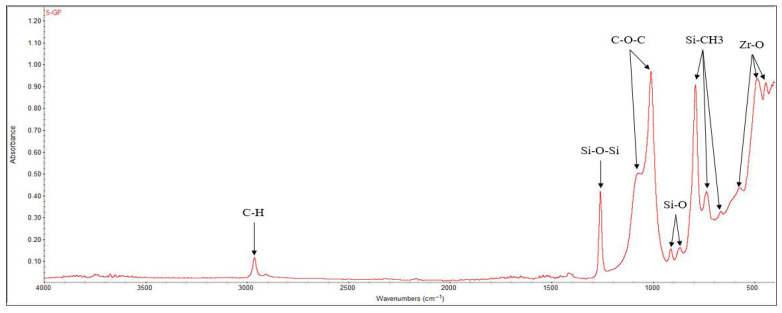
FT-IR spectra recorded for ROEKO GuttaFlow 2.

FT-IR analysis reveals the presence of chemical bonds corresponding to both organic and inorganic components of ROEKO GuttaFlow 2 (Figure 9). The bands located at 572 cm^−1^, 484 cm^−1^, and 443 cm^−1^ indicate Zr–O bonds, while the signals at 664 cm^−1^, 734 cm^−1^ and 789 cm^−1^ correspond to the vibrations of the Si–CH_3_ groups. The Si–O bonds are observed at 865 cm^−1^ and 910 cm^−1^. The most intense peak is attributed to the C–O–C (etheric) vibrations, detected at 1008 cm^−1^, while the Si–O–Si bond is highlighted at 1257 cm^−1^. Also, the presence of C–H groups is confirmed by the band at 2962 cm^−1^ [52,53].

#### 3.2.3. XRD Analysis

**Figure 10:**
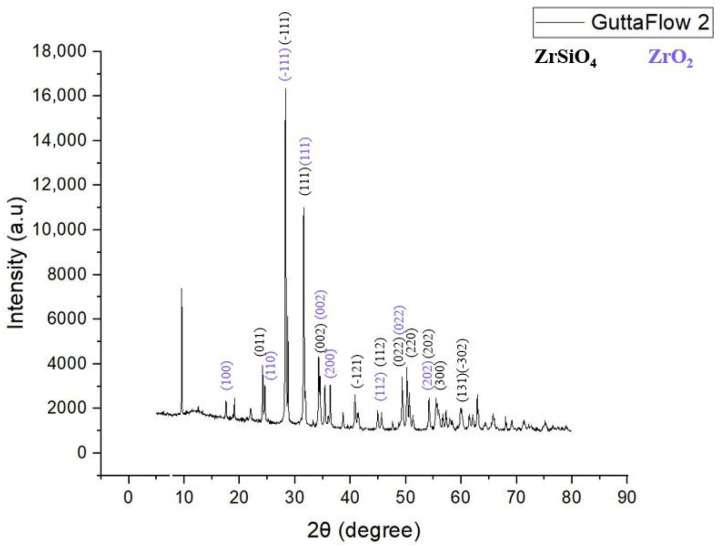
XRD analysis recorded for ROEKO GuttaFlow 2.

Through XRD analysis, two phases of the material were identified: zirconium silicate (ZrSiO_4_—ASTM 04-021-7113) [54] with a monoclinic structure, with the diffraction maximum positioned at the Miller index (−111), as the predominantly quantitative phase and zirconium oxide (ZrO_2_—ASTM 01-075-6808) [50] with monoclinic symmetry, as the secondary phase, with the diffraction maximum located at the Miller index (−111), according to ASTM data sheets (Figure 10). The maximum diffraction intensities, recorded at 16,360 and 2θ = 28.257° and 13,480 and 2θ = 28.309°, indicate a high degree of crystallinity in the material.

The correlations between ASTM reference data (hkl, d, 2θ, I%) and the experimentally measured 2θ and d-spacing are summarized in Table 4 and Table 5.

### 3.3. Metabolic Activity, Viability and Cytotoxicity Tests Results

All results were normalized to the untreated control group (set at 100%). The means of the control and test samples were calculated and reported relative to the control group at the experimental level. This procedure was applied to all metabolic activity, viability and cytotoxicity tests.

#### 3.3.1. MTT Assay

**Figure 11:**
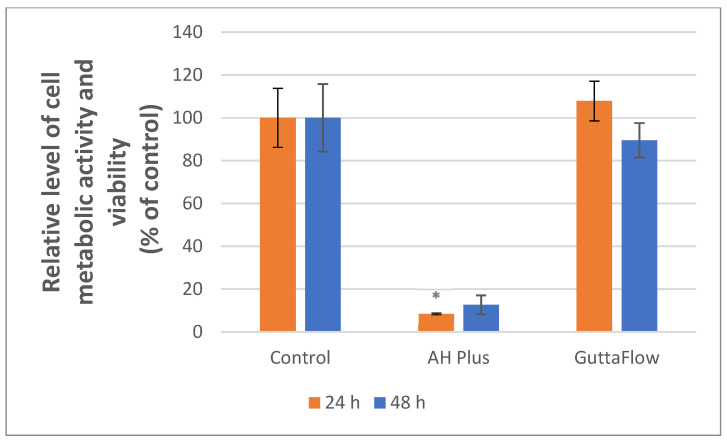
The metabolic activity and viability of the cells were evaluated following 24 and 48 h of exposure to AH Plus and ROEKO GuttaFlow 2. Unexposed cells served as the control. Values are illustrated as mean ± SD results (*n* = 3). For measurements at 24 h, Levene’s test, Welch’s ANOVA and Games–Howell post hoc test were applied (* *p* < 0.05). For 48 h, Kruskal–Wallis H and Mann–Whitney U post hoc tests, along with Bonfferoni correction, were applied. * indicate statistically significant differences compared to the control group.

To evaluate the biological activities of the two materials, the quantitative MTT test was performed on human osteosarcoma cells to assess their metabolic activity and viability. According to Figure 11, the MTT test indicated that the cell’s metabolic activity decreased significantly by ~92% at 24 h (* *p* < 0.05) and by ~87% after 48 h for the cells incubated with AH Plus material compared to the control sample. Regarding ROEKO GuttaFlow 2, metabolic activity and viability increase by ~8% in the first 24 h compared to the control. After 48 h, a reduction of MTT levels of ~11% was observed in cells exposed to ROEKO GuttaFlow 2. The drastic results recorded for the AH Plus material can be explained by the presence of cell-toxic components within the material, namely epoxy resins. The ISO 10993-5:2009 standard stipulates that materials that ensure a cell metabolic activity and viability greater than 70% can be considered biocompatible [55].

#### 3.3.2. LDH Assay

**Figure 12:**
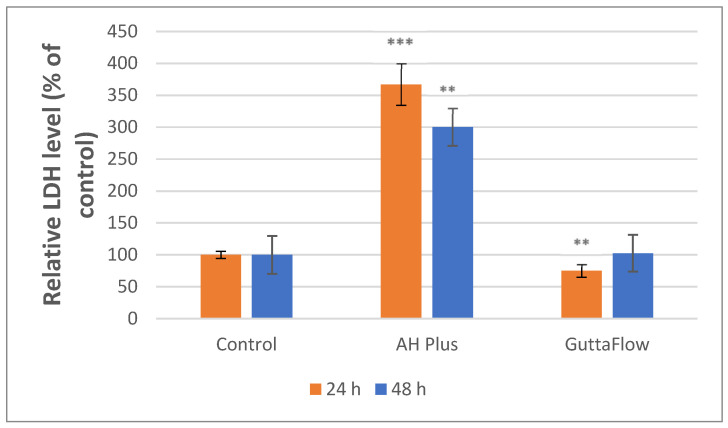
LDH level following 24 and 48 h of exposure to AH Plus and ROEKO GuttaFlow 2. Unexposed cells served as the control. Values are presented as mean ± SD results (*n* = 6). For measurements at 24 h, Levene’s test, Welch’s ANOVA and Games-Howell post-hoc test were applied (*p* < 0.01 **, *p* < 0.001 *). For 48 h, Kruskal–Wallis H and Mann–Whitney U post hoc tests, along with Bonferroni correction, were applied (*p* < 0.01 **). **, *** indicate statistically significant differences compared to the control group.

The cytotoxic effects have been quantified by assessing the released LDH levels, which were statistically significant in both analyzed samples. The results of the LDH test (Figure 12) showed a significant increase of ~267%, compared to the control, in the cells incubated with AH Plus after 24 h (*** *p* < 0.001). ROEKO GuttaFlow 2 exhibited a significant decrease in LDH level at 24 h (** *p* < 0.01). After 48 h of incubation, AH Plus showed a very high LDH value (~200%), which was statistically significant (** *p* < 0.01), while for ROEKO GuttaFlow 2, LDH levels increased by 2% compared to the control (Figure 12).

#### 3.3.3. NO Test

**Figure 13:**
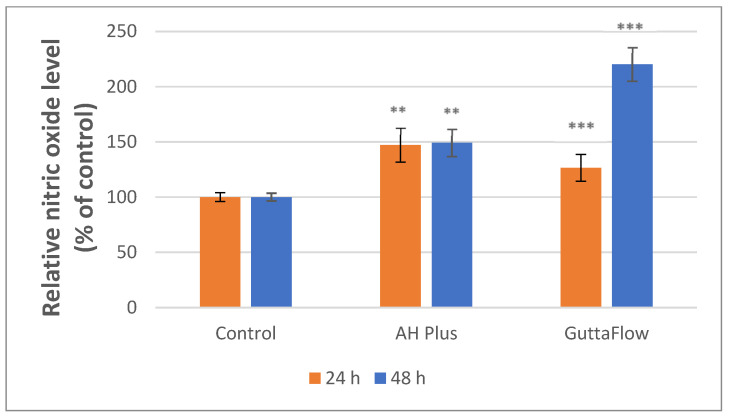
NO level following 24 and 48 h of exposure to AH Plus and ROEKO GuttaFlow 2. Unexposed cells served as the control. Values are presented as mean ± SD results (*n* = 6). For measurements at 24 h, Kruskal–Wallis H and Mann–Whitney U post hoc tests, along with Bonferroni correction, were applied (*p* < 0.01 **, *p* < 0.001 ***). For 48 h, Levene’s test, one-way ANOVA and Games–Howell post hoc test were applied (*p* < 0.01 **, *p* < 0.001 *). **, *** indicate statistically significant differences compared to the control group.

Regarding the Griess test, both materials showed statistically significant increases in NO levels after incubation of the cells with the materials compared to the control sample. After 24 h, the NO level increased significantly for both materials, by ~47% (** *p* < 0.01) for AH Plus and by ~26% (** *p* < 0.01) for ROEKO GuttaFlow 2, compared to the control. Moreover, after 48 h, the NO level for both materials continued to increase. Regarding AH plus, it increased significantly by approximately 50% (*** *p* < 0.001), while the silicone-based material, ROEKO GuttaFlow 2, showed a statistical increase of 120% (*** *p* < 0.001) compared to the control. Correlated with the results of the cytotoxicity test, the increased level of NO could be seen as an indicator of the adaptability of the incubated cells to the stress caused by contact with materials (Figure 13).

To improve data integration and comparative analysis, the results were organized in a tabular format (Table 6).

## 4. Discussion

When discussing patient safety and treatment success, an important starting point is the biocompatibility of the materials used. To assess the biological effects of these materials on the body, it is crucial, first, to test biocompatibility on cells in vitro. This enables the evaluation and measurement of their biological safety and potential [56].

Unlike other regions of the body, the oral cavity presents a series of challenges regarding both the success of treatments and the biocompatibility of materials due to its specific conditions and processes: the presence of saliva, creating a moist environment, as well as a high microbial load. In addition to meeting basic chemical and physical standards, materials used in the oral cavity must demonstrate increased viability and long-term durability in a humid environment [57,58,59].

To be regarded as an ideal endodontic material, it must exhibit antimicrobial activity against periodontal bacteria, provide a complete microscopic seal of the endodontic system, and be capable of achieving these factors without provoking an excessive inflammatory response in the host tissues or demonstrating cytotoxicity [60,61].

This study integrated microstructural, chemical, and biological analyses to compare two common endodontic sealers: an epoxy-resin-based one (AH Plus) and a silicone-based one (ROEKO GuttaFlow 2). The combined data demonstrate unique physicochemical structures that are linked to significantly different in vitro cytotoxicity profiles.

SEM analysis of AH Plus indicated a relatively irregular outer surface topography and a compact, homogeneous cross-section with inorganic polyhedral granules, containing Zr, Ca, and W, as suggested by elemental mapping, embedded within an epoxy resin matrix, 24 h after setting. After 14 days, both the outer surface of the sample and the interior changed their morphology: in both cases, the resin adopts a wavy pattern, which could indicate a setting contraction. This is undesirable, as it can facilitate microbial infiltration and multiplication at the tooth–resin interface, potentially compromising the root canal treatment [62,63]. Also, studies of the literature confirm the irregular surface topography of AH Plus samples [64]. Surface structure may significantly influence cell adhesion and proliferation. In some cases, this is not the decisive factor, but the composition of the surface might be more impactful [65,66,67]. Consequently, some studies have highlighted a low adhesion to the surface of the AH Plus material [64,68].

The energy-dispersive X-Ray spectrum revealed the main chemical elements of the material, listed in decreasing order of abundance: C, O, W, Zr, and Ca. The elements Si and N could not be identified by EDX either because of their low concentration in the material (Si) or due to the lack of sensitivity of this technique for light elements (N). However, FT-IR analysis identified Si-O and Si-CH bonds characteristic of silicone-based structures, along with N-H bands corresponding to nitrogen-containing (amine) groups, which are common in epoxy hardeners and curing agents [69]. Additionally, signals associated with tungstate-related functionalities were observed. XRD revealed the presence of two crystalline phases: tetragonal calcium tungstate (scheelite, CaWO_4_), as the primary phase, and monoclinic zirconium oxide (ZrO_2_), as a secondary phase. Overall, these findings suggest a ceramic-filled epoxy network with radiopacifiers made of heavy elements (W) and residual amine-based curing compounds [69].

Regarding ROEKO GuttaFlow 2, SEM analysis revealed a smooth, uniform outer surface, devoid of porosity. Such a surface may lead to an increased rate of cell adhesion [70,71]. A study conducted in 2014 on human periodontal ligament cell cultures (hPDLs) showed that, 2 h after letting the cells attach to ROEKO GuttaFlow 2, the attachment rate was very low compared to the control (*p* < 0.001). After rising, the cell attachment rate began to increase significantly, exceeding the control sample at 24 h [72]. In another study from 2017, also performed on hPDLs, the cells were incubated for 7 days in the presence of the material, with results indicating a modest attachment to ROEKO GuttaFlow 2 [73]. Inside, the material exhibits a heterogeneous silicone-based composite structure, consisting of an inorganic crystalline phase, mainly Zr-containing compounds, embedded within an organic silicone matrix. Pores and cavities related to the organic component were visible, and Zn appeared as distinct concentrations (1.16%). In dentistry, zinc, especially zinc oxide nanoparticles, has a wide range of applications in endodontics, as well as in periodontology, surgery, prosthetics, restorative dentistry, orthodontics, cancer diagnosis and preventive dentistry. In endodontics, the primary advantages of using zinc oxide nanoparticles are: antimicrobial effects, enhanced fracture resistance in roots and improved sealing ability [74,75,76]. According to a study carried out in 2018, a significant remineralization of the root dentin was observed, along with improved mechanical properties and excellent sealing of the teeth on which materials with nanoparticles of ZnO were used for endodontic fillings [77]. Another study demonstrated that gutta-percha cones coated with zinc nanoparticles exhibited antibacterial activity against E. faecalis and S. aureus, thus significantly reducing the chances of reinfection of the endodontic space [78]. Additionally, ZnO nanoparticles together with CaO, Na2O, P2O5 and SiO2 form a complex zinc bioglass, which can activate alkaline phosphatase (ALP), thereby stimulating the differentiation of pulp stem cells (hDPSCs) [79]. Memarzadeh et al. [80] conducted a study on osteoblasts (UMR-106 and MG-63), highlighting the fact that, upon exposure to different concentrations of ZnO-coated nanoparticles onto glass, the cell morphology did not change, but even provided a favorable substrate for adhesion, cell growth and metabolic activity.

According to the manufacturer’s data sheet [41], ROEKO GuttaFlow 2 also contains a platinum-based catalyst, an element that could not be identified by energy dispersive X-Ray spectroscopy, most likely due to the minimal quantity below the detection limit. The mapping provided valuable information about the distribution of elements, but also about the phases of the material, subsequently detected by XRD.

FT-IR analysis allowed the identification of the main functional groups present in the materials. The polydimethylsiloxane (PDMS) matrix of GuttaFlow2 showed characteristic absorption bands, including Si–O and Si–CH_3_ vibrations and broad Si–O–Si bending modes. In addition, bands corresponding to zirconium-containing compounds, such as ZrO_2_ and ZrSiO_4_, were observed in the “fingerprint” region, confirming their presence in the material. These results indicate that both the polymer matrix and the inorganic zirconium-based components contribute to the FT-IR spectrum, allowing a clear identification of the functional bonds and the “fingerprint” characteristics of the sealer. XRD identified zirconium silicate (ZrSiO_4_), of monoclinic symmetry, as the main crystalline phase, with a diffraction maximum at 16,360 near 28° along with secondary ZrO_2_, also monoclinic, with its maximum intensity at 12,257 around 28°.

The physicochemical features of the materials are closely linked to biological response, influencing cell behavior and overall biocompatibility. Overall, the biocompatibility tests showed a clear difference: AH Plus caused a significant drop in metabolic activity (over 90%, * *p* < 0.05 at 24 h) and a large significant increase in LDH release (~267%, *** *p* < 0.001 and ~200%, ** *p* < 0.01 for 24 and 48 h), indicating strong cytotoxicity under the tested conditions. In contrast, ROEKO GuttaFlow 2 kept cell metabolic activity near control levels for 24 h and only slightly decreased after 48 h. According to ISO 10993-5:2009 criteria, materials that maintain over 70% viability are considered non-cytotoxic. Based on this standard, ROEKO GuttaFlow 2 suggests substantially more biocompatible behavior than AH Plus in our study conditions.

Several material features probably account for these differences (Figure 1, Figure 2, Figure 4, Figure 5, Figure 6, Figure 7, Figure 9 and Figure 10). Epoxy resins and their amine hardeners are known sources of cellular toxicity and sensitization [81]. The FT-IR evidence showing C–N/N–H functionalities, along with a strong cytotoxic signature, supports this conclusion (Figure 11 and Figure 12). Polymerization shrinkage or incomplete curing could have increased exposure to unreacted toxic chemicals, aligning with the morphological signs of late shrinkage observed under SEM (Figure 1d–f).

Conversely, our results indicate that ROEKO GuttaFlow 2’s silicone matrix chemistry generally shows less cytotoxicity. Its filler components (ZrSiO_4_/ZrO_2_ and trace Zn) do not contain the reactive amine or epoxy groups typically associated with chemical stress and cell death. The trace Zn, known for its antimicrobial properties, might affect biological interactions, but at the concentrations tested, it did not cause significant cytotoxic effects [51].

Regarding AH Plus, Zhou et al. [82] indicated that the degree of cytotoxicity is directly proportional to the material’s concentration, while Silva et al. [83] have shown that freshly mixed AH Plus exhibits moderate cytotoxicity but becomes non-toxic after approximately one week of incubation with cells. In contrast, two studies that investigated ROEKO GuttaFlow 2, one performed on 3T3 fibroblasts, analyzing the MTT and LDH levels [84], and the other on human periodontal ligament stem cells (hPDLSCs) via an Annexin V/7-AAD test [85], also showed low cytotoxicity and high metabolic activity of this material. However, we were not able to find any studies targeting the response of G292 to ROEKO GuttaFlow 2.

Regarding nitric oxide responses and cell stress, both materials increased NO production compared to control, with ROEKO GuttaFlow 2 showing significant increases at 24 and 48 h (~26%, ** *p* < 0.01 and ~120%, *** *p* < 0.001). Moderate NO elevation must be interpreted alongside the results of the other two tests, MTT and LDH, thus being able to indicate a cellular stress or adaptive response or activation of inflammation and defense pathways [45,86]. The lack of significantly increased levels of LDH for ROEKO GuttaFlow 2 did not indicate metabolic impairment, suggesting that the NO increase likely reflects sublethal stress or adaptive activation, not outright cell death. In contrast, AH Plus caused significantly high NO levels at 24 and 48 h (** *p* < 0.01, *** *p* < 0.001), along with significant LDH release at both incubation times (*** *p* < 0.001, ** *p* < 0.01), and metabolic decline after 24 h (* *p* < 0.05), indicating an inflammatory response leading to cytotoxic cell damage.

Nitric oxide (NO) is a naturally occurring free radical with vital biological functions, including promoting angiogenesis, supporting vasodilation, wound healing, and tissue repair, as well as exhibiting antimicrobial, antitumoral, and antioxidant effects [87,88,89]. While the regenerative properties of nitric oxide (NO) in soft tissues are well established, its role in bone tissue repair has been less explored. Bone cells generate both constitutive and inducible forms of NO synthase (NOS), with cytokines such as TNF and IL-1 strongly stimulating NO production. NO’s effects on bone depend on its concentration: at low levels (pico- to nanomolar), it supports osteoblast proliferation, differentiation, and survival. Conversely, at higher concentrations (micromolar range), NO may inhibit bone resorption and formation. Therefore, within specific concentration ranges, NO can prevent osteoclast-driven bone resorption while promoting osteoblast growth [90,91,92].

Under the conditions of this in vitro study, these findings suggest the idea that silicone-based ROEKO GuttaFlow 2 has better biocompatibility than epoxy-based AH Plus when in direct contact with G292 cells in vitro. Since sealer components can be exposed to periapical tissues and bone through extrusion or leaching, it is preferable to choose materials that produce fewer cytotoxic leachates. However, clinical success also relies on factors such as sealing ability, dimensional stability, antimicrobial effectiveness, and long-term performance and on the proper execution of the root canal obturation technique by the operator. It is important to note that the materials are used in different obturation techniques. While AH Plus is applied together with gutta-percha cones, ROEKO GuttaFlow 2 can be used as a stand-alone sealer, without the need for cones, due to its chemical characteristics. Testing the biocompatibility and physicochemical properties of this material is justified by studies that have proposed root canal obturation techniques using only sealant, procedures that can be more time-efficient and relatively less expensive, without the need for specialized instruments [93]. Therefore, structural and cytotoxicity profiles should be considered alongside these other qualities when selecting sealers [24].

The study’s key strength is its integrated methodology, linking microstructural and compositional characterization with early in vitro assessment in G292 cells. At the same time, certain limitations establish the boundaries for data interpretation. First of all, the study employed a single cell line (G292 human osteosarcoma cells); cellular responses may vary across different cell types, such as bone marrow-derived mesenchymal stem cells, primary osteoblasts, periodontal ligament cells, macrophage-lineage cells or fibroblasts, and extend to biological endpoints other than nitric oxide release and metabolic activity. Second, the assays evaluated only early time points of incubation (24–48 h); longer-term leachate studies and chronic exposure assays would better characterize sustained biocompatibility because the material remains in the oral cavity for a long period of time. Third, a quantitative correlation between specific chemical species (e.g., residual monomers, specific amines, Zn concentrations) and biological effects was not performed; targeted chemical eluate analysis would clarify causative agents. Fourthly, direct-contact exposure may accentuate early chemical effects; moreover, in vitro biocompatibility cytotoxicity results are highly dependent on the experimental setup, and ISO 10993-5:2009-based testing frameworks [55,94] can produce variable outcomes between laboratories and testing configurations.

Future work should (1) analyze sealer eluates chemically (GC-MS/LC-MS) to identify and quantify leachable toxicants; (2) expand cytotoxicity testing and cell proliferation to primary human cells relevant to periapical tissues (periodontal ligament cells, fibroblasts, osteoblasts) and include longer time points and inflammatory cytokine profiling; (3) perform in vivo biocompatibility and tissue response studies; (4) address mechanical and sealing performances, as they remain important for supporting clinical applicability; and (5) correlate cytotoxicity with polymerization degree and curing kinetics to determine whether modified formulations or curing protocols can mitigate AH Plus-associated cytotoxicity [59,60,64,65,72,73,74,75,95].

**Figure 14:**
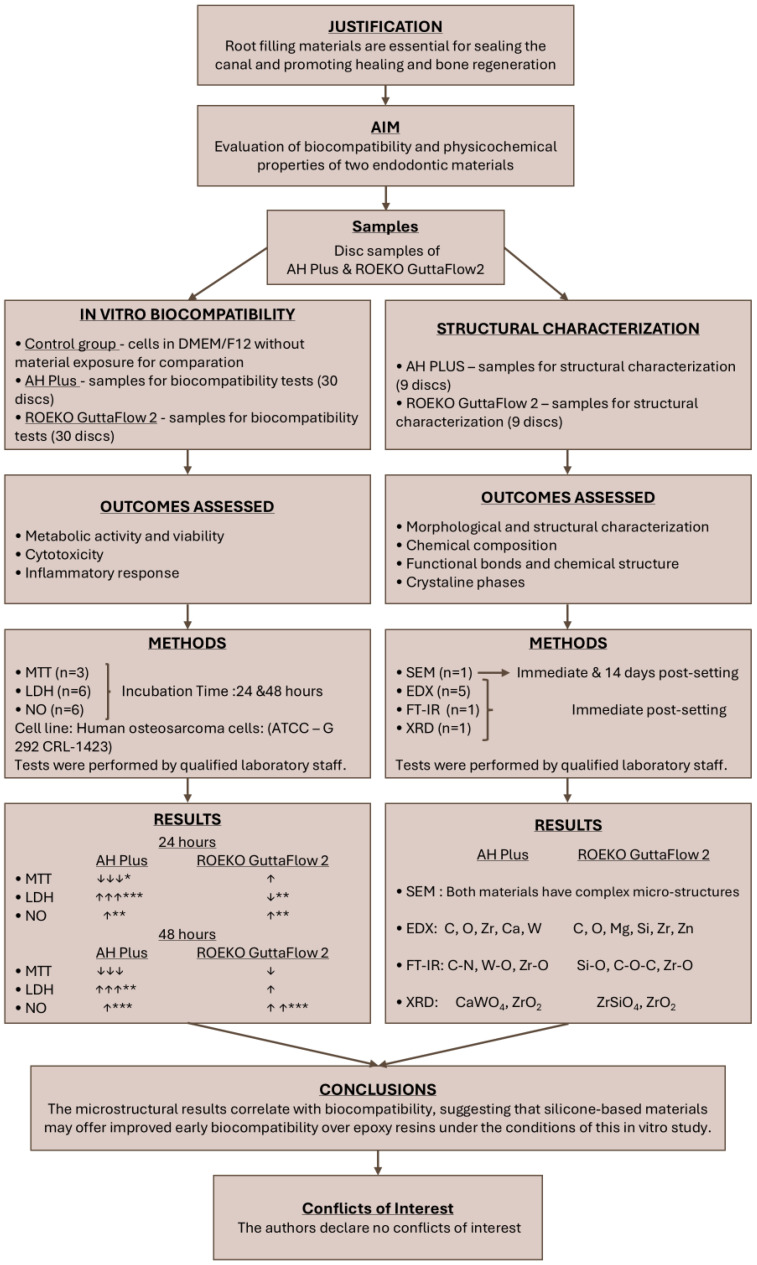
Flowchart illustrating the study’s workflow, following PRILE guidelines. Upward (↑) and downward (↓) arrows indicate percentage increases and decreases compared to the control (* *p* < 0.05, ** *p* < 0.01, *** *p* < 0.001) [96].

The study’s workflow, including the key steps, applied methods, and main results, is illustrated in a flowchart created in accordance with the PRILE guidelines [96]. This visual summary provides a clear and concise overview of the research process, facilitating a better understanding of the study’s progression and outcomes (Figure 14).

## 5. Conclusions

AH Plus and ROEKO GuttaFlow 2 exhibit distinct microstructural and chemical profiles that correlate with markedly different in vitro biological behaviors. ROEKO GuttaFlow 2 showed limited cytotoxicity and only modest stress signaling, whereas AH Plus induced pronounced cytotoxic effects consistent with epoxy/amine-related toxicity. These findings underscore the importance of comprehensive physicochemical characterization alongside biological testing when evaluating endodontic sealers and suggest that silicone-based formulations may offer improved early biocompatibility compared with the epoxy resin formulation tested under the conditions of this in vitro study.

## Figures and Tables

**Table 1:** The composition of the materials given by the manufacturers [40,41].

Material	Manufacturer	Lot Numbers	Material Type	Setting Time	Composition	
**AH Plus**	Dentsply Sirona DeTrey GmbHb	Paste A: 2506000016 Paste B: 2506000184	Epoxy resin	24 h	**Epoxide paste (paste A)**	**Amine paste** **(paste B)**
	De-Trey-Str.1 78467 Konstanz, Germany				Diepoxide	1-adamantane amine
					Calcium tungstate	N, N’-dibenzyl-5-oxa-nonandiamine-1,9
					Zirconium oxide	TCD-Diamine
					Aerosil	Calcium tungstate
					Pigment	Zirconium oxide
						Aerosil
						Silicone oil
**ROEKO** **GuttaFlow 2**	Coltene/Whaledent AG, Altstätten, Switzerland	M80914	Silicone-based	30 min	Gutta-percha powder Polydimethylsiloxane Platinum catalyst Zirconium dioxide Pigments	

**Table 2:** Comparison of ASTM 01-083-5705 reference data (hkl, d, 2θ, I%) with experimental XRD data of AH Plus.

CaWO_4_ (ASTM 01-083-5705)				AH Plus	
hkl	d	2 θ	I%	d	2 θ
101	4.75490	18.646	55.5	4.73753	18.715
112	3.10130	28.763	100	3.09710	28.803
103	3.06760	29.086	21.5	3.06462	29.115
004	2.83900	31.487	14.9	2.83720	31.507
200	2.61800	34.223	16.6	2.61503	34.263
211	2.29340	39.252	13.6	2.29175	39.281
204	1.92460	47.186	25.7	1.92464	47.185
220	1.85120	49.178	13.2	1.85089	49.187
116	1.68520	54.400	13.2	1.68557	54.387
312	1.58960	57.971	20.9	1.59014	57.949

**Table 3:** Comparison of ASTM 01-075-6808 reference data (hkl, d, 2θ, I%) with experimental XRD data of AH Plus.

ZrO_2_ (ASTM 01-075-6808)				AH Plus	
hkl	d	2 θ	I%	d	2 θ
−111	3.16210	28.199	100	3.09984	28.777
111	2.84160	31.457	68.1	2.83491	31.533
002	2.62710	34.101	20.6	2.61695	34.237
−211	2.21210	40.757	13.5	2.25171	40.009
022	1.84840	49.258	17.9	1.84997	49.213
202	1.69620	54.018	11.2	1.68631	54.361

**Table 4:** Comparison of ASTM 04-021-7113 reference data (hkl, d, 2θ, I%) with experimental XRD data of ROEKO GuttaFlow 2.

ZrSiO_4_ (ASTM 04-021-7113)			ROEKO GuttaFlow 2		
hkl	d	2 θ	I%	d	2 θ
011	3.69410	24.071	19.7	3.69417	24.149
−111	3.16560	28.167	100	3.16559	28.257
111	2.83710	31.508	60.7	2.83712	31.559
002	2.62440	34.137	17.6	2.62440	34.237
−121	2.17840	41.416	7.4	2.17842	41.439
112	2.01890	44.859	10.9	2.01889	44.897
022	1.84710	49.295	18.6	1.84709	49.343
220	1.81710	50.164	23.1	1.81711	50.175
202	1.69270	54.139	11.9	1.69270	54.179
300	1.69270	54.139	11.9	1.69270	54.179
131	1.54290	59.901	15.1	1.54291	59.847
−302	1.54290	59.901	15.1	1.54291	59.873

**Table 5:** Comparison of ASTM 01-075-6808 reference data (hkl, d, 2θ, I%) with experimental XRD data of ROEKO GuttaFlow 2.

ZrO_2_ (ASTM 01-075-6808)				ROEKO GuttaFlow 2	
hkl	d	2 θ	I%	d	2 θ
100	5.07960	17.445	6.0	5.05073	17.545
110	3.63440	24.473	11.8	3.62475	24.539
−111	3.16210	28.199	100	3.15001	28.309
111	2.84160	31.457	68.1	2.83720	31.507
002	2.62710	34.101	20.6	2.61888	34.211
200	2.53980	35.311	15.3	2.53491	35.381
112	2.02260	44.772	7.1	2.01616	44.923
022	1.84840	49.258	17.9	1.84358	49.395
202	1.69620	54.018	11.2	1.69455	54.075

**Table 6:** Variation of the evaluated parameters as % of control (% of control, mean ± SD, * *p* < 0.05; ** *p* < 0.01; *** *p* < 0.001).

Incubation Time		24 h		48 h	
Material/Test	Control	AH Plus	ROEKO GuttaFlow 2	AH Plus	ROEKO GuttaFlow 2
MTT	100%	8.59 (±0.38)% *	107.83 (±9.29)%	12.67 (±4.35)%	89.50 (±8.05)%
LDH	100%	366.81 (±32.89)% ***	74.84 (±10.07)% **	300.21 (±29.41)% **	102.34 (±28.81)%
NO	100%	147.18 (±15.37)% **	126.50 (±12.17)% **	149.13 (±12.50)% ***	220.23 (±15.34)% ***

## Data Availability

The original contributions presented in this study are included in the article/Supplementary Material. Further inquiries can be directed to the corresponding author(s).

## Supplementary Materials

The following supporting information can be downloaded at: https://www.mdpi.com/article/10.3390/ma19071388/s1.

## Abbreviations

The following abbreviations are used in this manuscript:

TNF-α	Tumoral necrosis factor alpha
IL-1β	Interleukin-1 beta
IL-6	Interleukin-6
IL-8	Interleukin-8
MIP-1	Macrophage inflammatory protein-1
MCP-1	Monocyte chemoattractant protein-1
RANKL	Receptor activator of nuclear factor-κB ligand
NO	Nitric oxide
G292	Human osteosarcoma cells
SEM	Scanning electron microscopy
EDX	Energy dispersive X-Ray
FEG	Field-emission electron gun
FT-IR	Fourier-transform infrared
XRD	X-Ray diffraction
3D	Three-dimensional
DMEM/F12	Dulbecco’s modified Eagle medium
MTT	3-(4,5-dimethylthiazol-2-yl)-2,5-diphenyltetrazolium bromide
LDH	Lactate dehydrogenase
NADPH	Nicotinamide adenine dinucleotide phosphate
SD	±Standard deviation
SPSS	Statistical Package for the Social Sciences
CaWO4	Calcium tungstate/scheelite
ZrO_2_	Zirconium oxide
ZrSiO_4_	Zirconium silicate
ASTM	American Society for Testing and Materials
hPDLs	Human periodontal ligaments
ALP	Alkaline phosphatase
hDPSCs	Human dental pulp stem cells
UMR-106	Rat osteosarcoma cell line
MG-63	Human osteosarcoma cell line
hPDLSCs	Human periodontal ligament stem cells
NOS	Nitric oxide synthase
GC-MS	Gas chromatography–mass spectrometry
LC-MS	Liquid chromatography–mass spectrometry
