# Supplementary material for: Physicochemical Characterization and In Vitro Biocompatibility of Epoxy- and Silicone-Based Endodontic Materials
Source: Materials (Basel). 2026 Mar 31;19(7):1388. doi: 10.3390/ma19071388 (PMC13074519; doi:10.3390/ma19071388)
Supplement: Supplementary file 1 [file materials-19-01388-s001.zip › materials-4151414-supplementary.pdf]

AH Plus

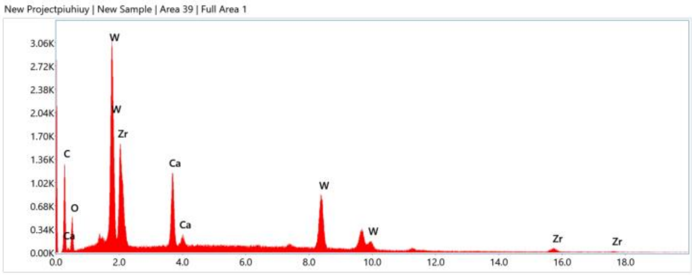

Element	Weight %	Atomic %
C K	45.2	75.7
O K	13.3	16.7
Ca K	4.1	2.1
Zr L	12.7	2.8
W L	24.6	2.7

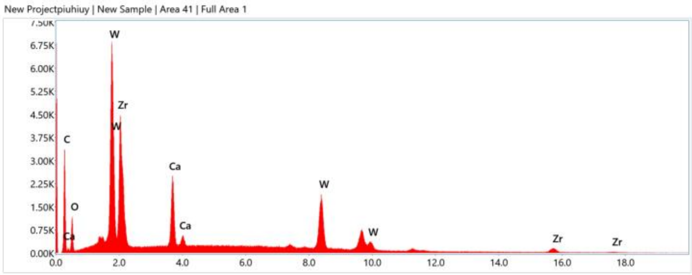

Element	Weight %	Atomic %
C K	48.3	78.3
O K	12.1	14.7
Ca K	3.5	1.7
Zr L	13.8	2.9
W L	22.3	2.4

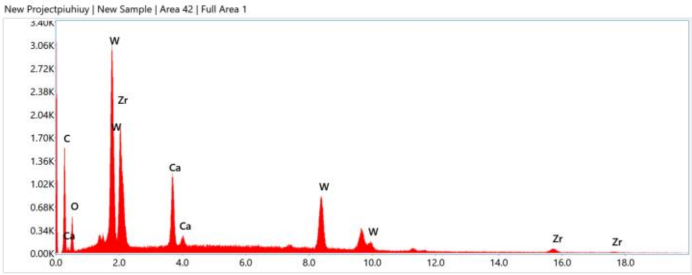

Element	Weight %	Atomic %
C K	48.6	78.6
O K	11.9	14.5
Ca K	3.7	1.8
Zr L	13.0	2.8
W L	22.8	2.4

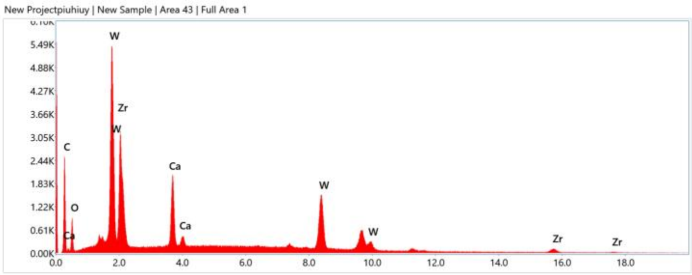

Element	Weight %	Atomic %
C K	46.9	77.8
O K	11.8	14.7
Ca K	3.9	1.9
Zr L	13.0	2.8
W L	24.4	2.7

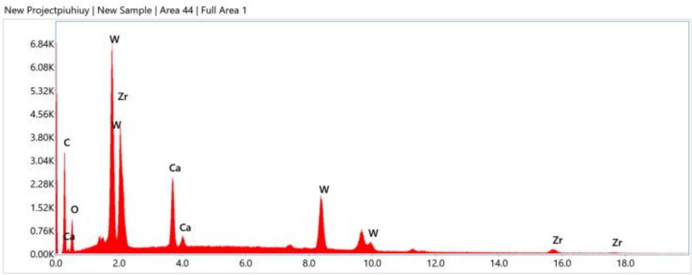

Element	Weight %	Atomic %
C K	48.2	78.6
O K	11.6	14.2
Ca K	3.7	1.8
Zr L	13.3	2.9
W L	23.1	2.5

Element	Weight %	Atomic%
C K	47.44 ± 1.41%	77.80 ± 1.22%
O K	12.14 ±0.67%	14.96 ± 0.99%
Ca K	3.78 ± 0.23%	1.86 ± 0.15%
Zr L	13.16 ± 0.42%	2.84 ± 0.05%
W L	23.44 ± 1.01%	2.54 ± 0.15%

ROEKO GuttaFlow 2

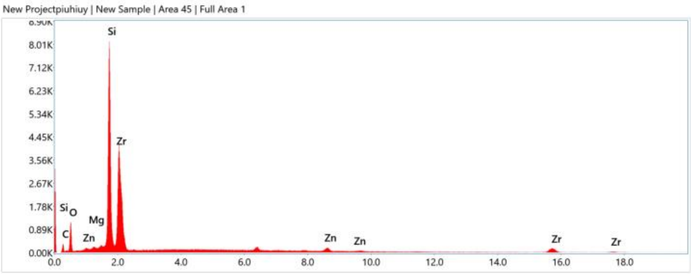

Element	Weight %	Atomic %
C K	21.7	38.6
O K	30.0	40.0
Mg K	0.5	0.5
Si K	18.4	14.0
Zn K	1.0	0.3
Zr L	28.4	6.7

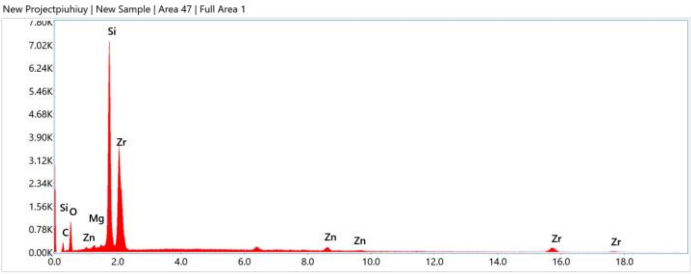

Element	Weight %	Atomic %
C K	22.1	39.0
O K	30.2	39.9
Mg K	0.6	0.5
Si K	18.4	13.9
Zn K	1.0	0.3
Zr L	27.7	6.4

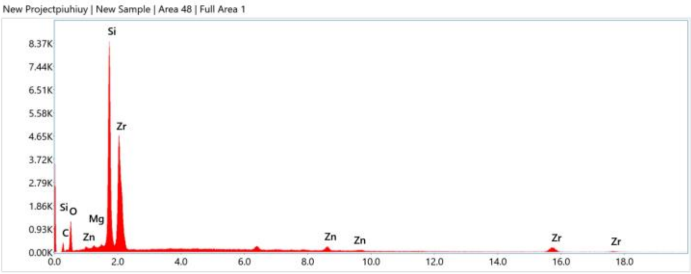

Element	Weight %	Atomic %
C K	20.9	37.8
O K	29.8	40.5
Mg K	0.4	0.4
Si K	17.8	13.8
Zn K	1.1	0.4
Zr L	29.9	7.1

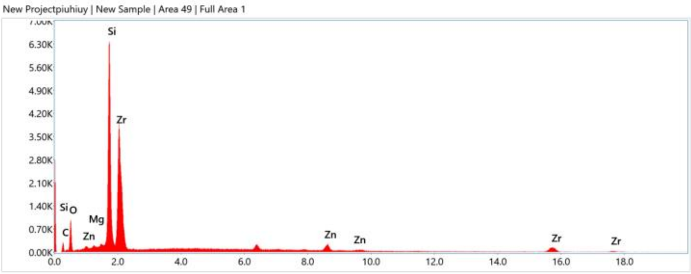

Element	Weight %	Atomic %
C K	20.7	37.8
O K	29.4	40.3
Mg K	0.4	0.4
Si K	17.3	13.5
Zn K	1.6	0.5
Zr L	30.6	7.4

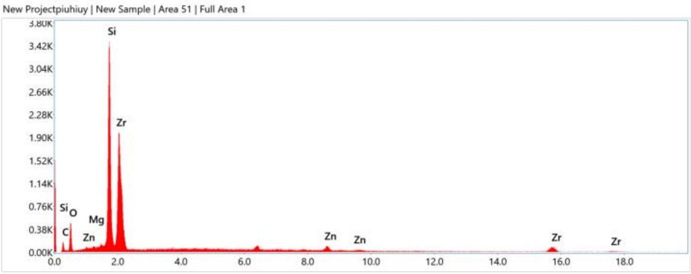

Element	Weight %	Atomic %
C K	24.0	42.2
O K	28.4	37.5
Mg K	0.4	0.4
Si K	17.1	12.9
Zn K	1.1	0.4
Zr L	29.0	6.7

Element	Weight %	Atomic%
C K	21.88 ± 1.32%	39.08 ± 1.82%
O K	29.56 ± 0.71%	39.64 ± 1.22%
Mg K	0.46 ± 0.09%	0.44 ± 0.05%
Si K	17.80 ±0.60%	13.62 ± 0.44%
Zn K	1.16 ± 0.25%	0.38 ± 0.08%
Zr L	29.12 ± 1.16%	6.86 ± 0.39%
